# Virgin Coconut Oil Associated with High-Fat Diet Induces Metabolic Dysfunctions, Adipose Inflammation, and Hepatic Lipid Accumulation

**DOI:** 10.1089/jmf.2019.0172

**Published:** 2020-07-09

**Authors:** Deise Jaqueline Ströher, Micaela Federizzi de Oliveira, Patrícia Martinez-Oliveira, Bruna Cocco Pilar, Márcia Denise Pavanelo Cattelan, Eliseu Rodrigues, Kalyne Bertolin, Paulo Bayard Dias Gonçalves, Jacqueline da Costa Escobar Piccoli, Vanusa Manfredini

**Affiliations:** ^1^ Graduate Program in Biochemistry, Federal University of Pampa, Uruguaiana, Brazil.; ^2^ Course of Pharmacy, Federal University of Pampa, Uruguaiana, Brazil.; ^3^ Graduate Program of Pharmaceutical Sciences, Federal University of Pampa, Uruguaiana, Brazil.; ^4^ Institute of Food Science and Technology, Federal University of Rio Grande do Sul, Porto Alegre, Brazil.; ^5^ Biotechnology and Animal Reproduction Laboratory, Federal University of Santa Maria, Santa Maria, Brazil.; ^6^ Graduate Program of Physiological Sciences, Federal University of Pampa, Uruguaiana, Brazil.

**Keywords:** coconut oil, metabolism, saturated fatty acids

## Abstract

Obesity reaches an epidemic level worldwide, and this condition is associated with chronic low-grade inflammation and secondary comorbidities, largely driven by global changes in lifestyle and diet. Various dietary approaches are proposed for the obesity treatment and its associated metabolic disorders. Good taste, antioxidant functions, and vitamins have been attributed to virgin coconut oil (VCO). However, VCO contains a large amount of saturated fatty acids, and the consumption of this fat is associated with a number of secondary diseases. We evaluate the effects of VCO supplementation on biochemical, inflammatory, and oxidative stress parameters in rats fed with high-fat diet (HFD). After feeding with HFD for 12 weeks, the animals were supplemented with VCO for 30 days. HFD+VCO group increased in diet intake, weight gain, low-density lipoprotein cholesterol level, and aspartate aminotransferase (AST) and alanine aminotransferase (ALT) levels. These findings were accompanied by increased in hepatic lipid profile and fat deposition in the liver. Adipocyte hypertrophy was observed in the HFD+VCO group, which was associated with elevated expression of tumor necrosis factor alpha (TNF-*α*) in adipose tissue. These results revealed that VCO associated with HFD induced important metabolic alterations, adipose inflammation, and hepatic lipid accumulation in rats.

## Introduction

Obesity is associated with chronic low-grade inflammatory state and is an underlying condition for inflammatory and metabolic diseases.^1^ There are many contributing factors to overweight and obesity, but diet is still the most important to develop this condition.^2^ The most commonly used strategies for the treatment of obesity and secondary diseases are low-fat diets, physical activity and pharmacotherapy.^3,4^ Nonetheless, pharmacotherapy is often associated with adverse effects.^4,5^ In this regard, it has been seeking some dietary compounds such as polyphenols and certain fatty acids that have potential to improve the obesity-associated metabolic disorders.^6^

Virgin coconut oil (VCO) has become popular and is gaining recognition, including among the scientific community,^7^ due to its beneficial properties and its usefulness as a functional food oil.^8^ The pharmacological benefits of VCO have been attributed to its high polyphenol content, such as ferulic acid, vanillic acid, syringic acid, quercetin, and caffeic acid.^9,10^ However, VCO is the unrefined oil obtained from the fresh coconut kernel, which is rich in saturated fatty acids (SFA; about 90%), such as lauric acid (C12:0), medium-chain fatty acids (MCFA),^9,11^ and tocopherol.^12^

SFA intake is associated with a number of secondary diseases, such as insulin resistance, glucose intolerance, dyslipidemia, cardiovascular disease (CVD), and hepatic steatosis.^13–15^ Although the evidence is pointing to the benefits of VCO, the most recent recommendations suggests limiting saturated fats.^16^ Furthermore, the 2017 American Heart Association Presidential advisory on dietary fats highlighted the paucity of evidence over the long-term health effects of SFA, such as coconut oil, and reinforced strongly recommendations to lower dietary saturated fat.^17^

The use of coconut oil is promoted by the media for weight loss, improvement of lipid profile, and others health effects, but most studies evaluated the effects of this oil associated with a normal diet.^18–21^ Considering the negative effects of a high-fat diet (HFD) and the recent recommendations on the consumption of SFA, we investigate the effects of VCO associated with HFD on biochemical, inflammatory, and oxidative stress parameters.

## Materials and Methods

### Virgin coconut oil

The VCO used in this study was obtained from Mundo dos Óleos (Brasília, DF, Brazil). According to the manufacturer's information, it was obtained by cold pressing and filtration from fruit. We analyzed the fatty acid profile in the VCO before using it, and the results are in Table 1. VCO contained MCFA as the dominant fatty acids. Lauric acid was the most abundant MCFA (53.6%). Myristic acid was the second major fatty acid in VCO sample (18.8%). Palmitic acid (10.7%), capric acid (5.4%), oleic acid (4.6%), butyric acid (3.7%), estearic acid (1.8%), and linoleic acid (1.1%) also were identified.

**Table 1.** Profile of Fatty Acids Methyl Esters of the Virgin Coconut Oil

Fatty acid	Virgin coconut oil
Butyric acid (C4:0)	3.7 ± 0.5
Capric acid (C10:0)	5.4 ± 0.4
Lauric acid (C12:0)	53.6 ± 0.4
Myristic acid (C14:0)	18.8 ± 0.1
Palmitic acid (C16:0)	10.7 ± 0.6
Estearic acid (C18:0)	1.8 ± 0.2
Oleic acid (C18:1)	4.6 ± 0.2
Linoleic acid (C18:2)	1.1 ± 0.0

Values expressed as mean ± SD.

SD, standard derivation.

### Determination of fatty acid methyl esters

The fatty acid profile of VCO oil was determined as fatty acid methyl esters (FAMEs), according to the previously reported method,^22^ using gas chromatography (CG Model 2010; Shimadzu, Kyoto, Japan) and capillary column (30 m × 0.25 mm, and 0.25 mm). The chromatographic conditions were previously described by Menegol *et al*.^23^

### Animals and diet

Male 30-day-old Wistar rats were purchased from the Central Animal Laboratory of the Federal University of Santa Maria (RS/Brazil). The rats were housed four per cage at a constant room temperature, humidity, and light cycle (12 h:12 h; light:dark) with free access to tap water and fed with chow *ad libitum*. This study was carried out in strict accordance with the recommendations in the NIH Guide for the Care and Use of Laboratory Animals and the local Institution Animal Care and Use Committee (Protocol No. 042/2015).

After adaptation period, the animals were weighed and they began to eat the experimental diet. The HFD consisted of commercial rat chow associated with lard-based (16 g of lard/200 g of rat chow) was given fresh each day. The commercial rat chow achieved, on average, 22.5% protein, 8.7% fat, 41% carbohydrate, and 3.7% fiber. The purchase lard has about 39 g saturated fat, 45 g monounsaturated fat, 11 g polyunsaturated fat, and 95 mg of cholesterol/100 g of lard. The nutritional content of the diet, after the addition of lard, achieved, on average, 20.8% protein, 15.4% fat, 37.9% carbohydrate, and 3.4% fiber. Based on these values, 100 g of the HFD offered 1502 kJ. Food consumption was measured daily by weighing uneaten food each morning, at the same time.

### Experimental design

Twelve rats were divided into two groups, each one containing six animals. After 12 weeks of diet, the animals were supplemented by gavage for 30 consecutive days, as follows: Group 1 (HFD): 2 mL/kg body weight of saline and Group 2 (HFD+VCO): 2 mL/kg body weight of VCO. At the end of the experiment, after 12 h fasting, the animals were anesthetized and blood samples were collected through cardiac puncture. Aorta, liver, and adipose tissue were collected for histological analysis. The retroperitoneal and epididymal fat tissues were weighted and frozen in liquid nitrogen for RNA extraction.

### Biochemical analysis

Serum concentration of total cholesterol, high-density lipoprotein (HDL) cholesterol, low-density lipoprotein (LDL) cholesterol, total triglycerides, glucose, alanine aminotransferase (ALT), and aspartate aminotransferase (AST) were performed using the ChemWell Automated Analyzer (Awareness Technology, Inc., Palm City, FL, USA) via colorimetric methods using commercial kits (Labtest^®^; Diagnostica).

### Analysis of lipid profile and oxidative damage in hepatic tissue

#### Extraction and quantification of total liver fat

The total liver fat was extracted and quantified as previously described.^24^ The hepatic tissue was macerated, and chloroform and methanol were added. Subsequently, the sample was filtered into a test tube and distilled water was added to it. The test tube was shaken and centrifuged for phase separation. The aliquot of the lower phase was transferred to a beaker which was transported to an oven (temperature 60°C) until the chloroform was completely evaporated. At the end, the beaker was again weighed and the amount of total fat was obtained by the difference in weighing of the beaker related to the initial used mass of the hepatic tissue times the amount of chloroform used in the technique.

#### Determination of total cholesterol and hepatic triglycerides

After the extraction of the total liver fat, the obtained residue was resuspended with isopropyl alcohol and aliquots were removed for analysis. The levels of total cholesterol and triglycerides were performed by spectrophotometric methods, using commercial kits (Bioclin^®^, Belo Horizonte, MG, Brazil).

#### Evaluation of oxidative stress parameters

The lipid peroxidation^25^ and carbonyl protein^26^ were measured in the plasma, and liver by spectrophotometric methods. The evaluation of DNA damage was made through the analysis of the frequency of micronucleus^27^ in leukocytes.

Activities of superoxide dismutase (SOD; RANSOD Kit; RANDOX Brazil LTDA), catalase (CAT),^28^ and glutathione peroxidase (GPx; RANSEL Kit; RANDOX Brazil LTDA) were determined from erythrocytes samples.

#### Histological analysis

Fragments of liver, adipose tissues, and aortas were removed and fixed in 10% buffered formalin, dehydrated, and embedded in paraffin. Histological sections of the tissues were taken in a microtome at a thickness of 5 *μ*m and stained with hematoxylin and eosin. The aortic thickening was measurement with the LAS EZ-V 3.4.0 software. The sectional area of the adipocytes was measured on digital images (50 adipocytes/animal)^29^ and analyzed with Image-Pro Plus (Media Cybernetics, Silver Spring, MD, USA).

#### RNA isolation, reverse transcription, and real-time quantitative polymerase chain reaction

Total RNA from retroperitoneal and epididymal adipose tissues was extracted using TRIzol^®^. Quantification of RNA was performed using a Nano-Drop spectrophotometer. RNA was treated with 0.1 U DNase Amplification Grade (Invitrogen), followed by DNase inactivation with 1 *μ*L of EDTA. Double-stranded complementary DNA (cDNA) was synthetized from 300 ng of total RNA with random hexamer primers using iScript cDNA Synthesis Kit (Bio-Rad). Quantitative real-time polymerase chain reactions (qRT-PCR) were conducted in a CFX384 thermocycler (Bio-Rad) using BRYT Green^®^ dye and Taq DNA polymerase from GoTaq^®^ qPCR Master Mix (Promega Corporation), with 12.5 ng of cDNA in 10 *μ*L. Primers sequences are listed in Table 2. The results are expressed relative to GAPDH and YWHAZ levels. Data were then normalized to a calibrator sample using ΔΔCq method as previously described.^30^

**Table 2.** Genes Tested by Quantitative Real-Time Polymerase Chain Reaction Analysis and Used Forward and Reverse Primers

Genes	Primers sequences
Leptin (Lep)	F—AGCAGCTGCAAGGTCCAAG
	R—AGGGTTTTGGTGTCATCCTGG
Adiponectin (Adipoq)	F—CCACCCAAGGAAACTTGTGC
	R—GACCAAGAACACCTGCGTCT
Tumor necrosis factor alpha (TNF-*α*)	F—TCGGTCCCAACAAGGAGGAG
	R—CTCCGCTTGGTGGTTTGCTAC

### Statistical analysis

The statistical analysis was performed by using GraphPad Prism 5. The results were analyzed using the unpaired Student's *t*-test. The data are reported as mean ± standard derivation. Results were considered statistically significant when *P* < .05.

## Results

### Body weight, food intake, and biochemical parameters

At the end of the experiment, the animals from HFD+VCO group presented a significant increase in the body weight gain (*P* < .05), food intake (*P* < .0001), LDL cholesterol (*P* < .0001), and liver function enzymes (ALT—*P* < .0001 and AST—*P* < .05) when compared to those from HFD group. However, the association of VCO with HFD did not trigger increase of HDL cholesterol and cause lower levels of total cholesterol (*P* < .01) and glucose (*P* < .0001; Table 3).

**Table 3.** Effects of High-Fat Diet Consumption and Virgin Coconut Oil Supplementation on Weight Gain, Food Intake, and Biochemical Parameters

	HFD	HFD+VCO
Initial body weight (g)	87.2 ± 6.5	85.9 ± 5.9
Final body weight (g)	461.3 ± 30.1	494.7 ± 64.2
Body weight gain (g)	374.1 ± 29.3	408.8 ± 64.3^*^
Diet intake (g/day)	103.9 ± 20.2	116.3 ± 10.8^***^
Total cholesterol (mg/dL)	106.2 ± 16.6	96.4 ± 10.6^**^
Triglyceride (mg/dL)	121.0 ± 22.1	110.4 ± 21.8
HDL-cholesterol (mg/dL)	22.2 ± 3.8	20.6 ± 2.6
LDL-cholesterol (mg/dL)	108.4 ± 10.5	139.7 ± 17.3^***^
Glucose (mg/dL)	253.4 ± 18.2	180.8 ± 30.3^***^
Alanine aminotransferase (U/L)	94.4 ± 33.7	156.2 ± 39.0^***^
Aspartate aminotransferase (U/L)	144.2 ± 28.0	171.8 ± 52.8^*^

Data are expressed as mean ± SD.

^*^ *P* < .05, ^**^P < .01, ^***^P < .0001 versus HFD group.

HDL, high-density lipoprotein; HFD, high-fat diet; HFD+VCO, high-fat diet+virgin coconut oil; LDL, low-density lipoprotein.

### Lipid profile and oxidative damage in hepatic tissue

**FIG. 1.**
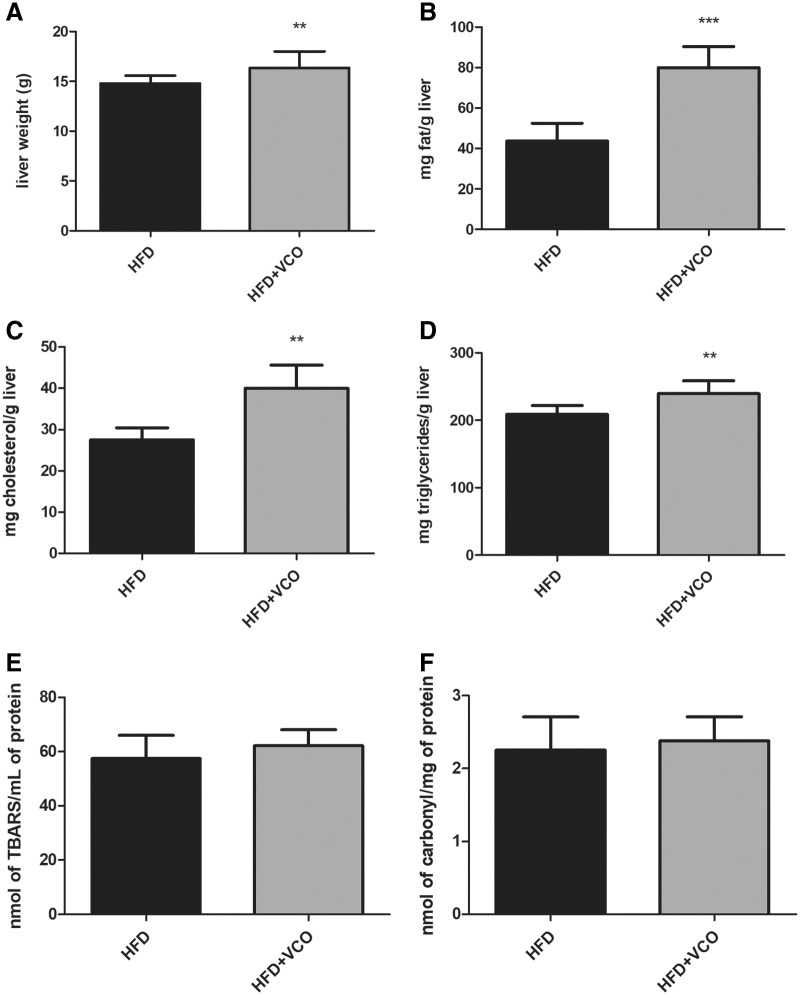
Effects of HFD consumption and VCO supplementation on liver weight, total liver fat, hepatic lipid profile, and hepatic oxidative damage. In **(A)** liver weight; **(B)** total liver fat; **(C)** total hepatic cholesterol; **(D)** hepatic triglycerides; **(E)** lipid peroxidation levels; **(F)** carbonyl protein contents. Data are expressed as mean ± SD. **P < .01, ***P < .0001 versus HFD group. HFD, high-fat diet; SD, standard derivation; VCO, virgin coconut oil.

Hepatic tissue weight (*P* < .01), total liver fat (*P* < .0001), total cholesterol (*P* < .01), and triglycerides (*P* < .01) were higher in animals from HFD+VCO group than those from the HFD group (Fig. 1A–D). On the contrary, lipid peroxidation and protein carbonylation did not differ between groups in the hepatic tissue (Fig. 1E, F).

### Oxidative stress parameters and antioxidant defenses

**FIG. 2.**
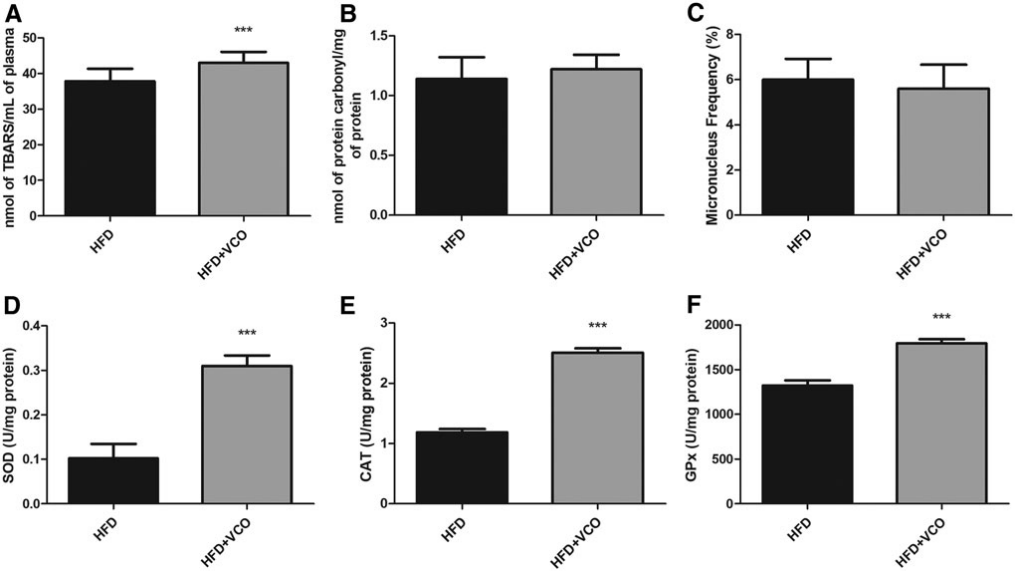
Effects of HFD consumption and VCO supplementation on biomarkers of plasma oxidative damage and antioxidant defenses. In **(A)** lipid peroxidation (TBARS); **(B)** protein carbonyl levels; **(C)** micronucleus test; **(D)** SOD; **(E)** CAT; and **(F)** GPx. Data are expressed as mean ± SD. ***P < .0001 versus HFD group. CAT, catalase; GPx, glutathione peroxidase; SOD, superoxide dismutase.

The association of VCO with HFD induced an increase in lipid peroxidation (Fig. 2A, *P* < .0001) compared with HFD group. Evaluation of the protein carbonylation and micronucleus frequency revealed no differences between groups (Fig. 2B, C). However, VCO triggered an increase in the antioxidant defense (SOD activity—*P* < .0001; Fig. 2D); CAT—*P* < .0001; Fig. 2E) and GPx—*P* < .0001; Fig. 2F) compared with HFD group.

### Histological analysis

**FIG. 3.**
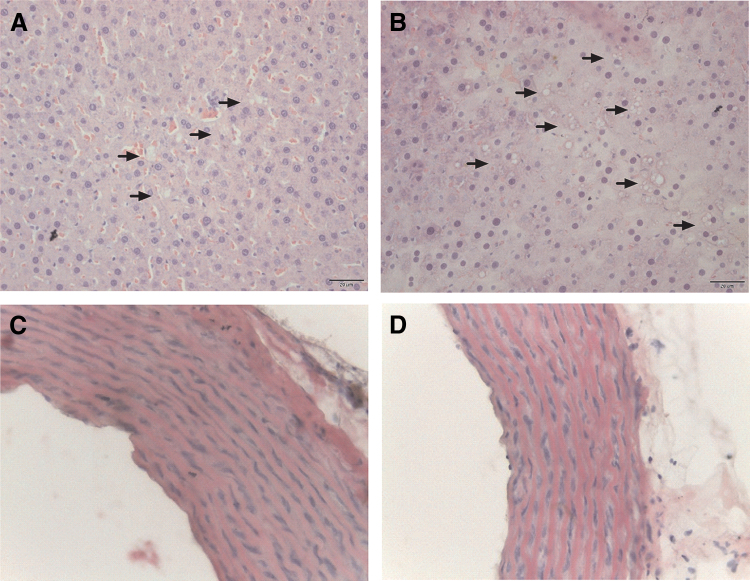
Photomicrographs of histological sections of livers **(A, B)** and aorta **(C, D)** from rats fed a HFD without or with VCO supplementation, respectively. Serial sections of the liver and aorta specimens were stained with hematoxylin and eosin (40 × ). *Arrows* show lipids droplets deposition in livers from HFD and HFD+VCO groups. The aortic intima-media thickness was evaluated and differences were not observed. Color images are available online.

Liver histological evaluation revealed that the association of VCO with HFD cause a higher number of lipids droplets (Fig. 3B) when compared with HFD without VCO (Fig. 3A), but did not change aortic intima-media thickness (IMT) (Fig. 3C, D).

**FIG. 4.**
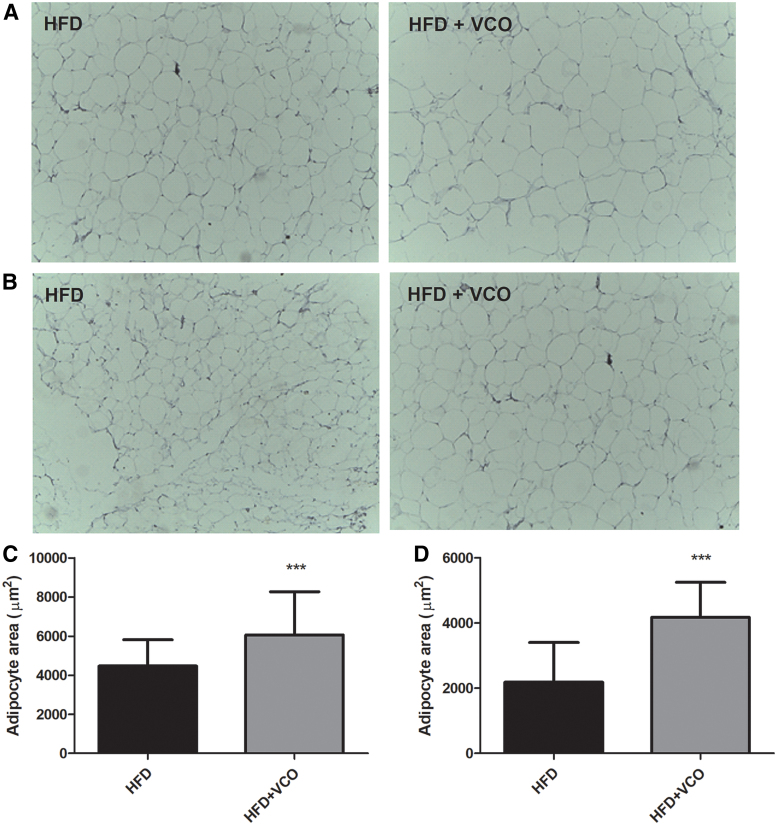
Effects of HFD consumption and VCO supplementation on area of adipocytes in retroperitoneal and epididymal adipose tissues. Serial sections of the specimens were stained with hematoxylin and eosin (40 × ). In **(A)** retroperitoneal adipose tissue; in **(B)** epididymal adipose tissue. In **(C)** area of adipocytes in retroperitoneal adipose tissue; in **(D)** area of adipocytes in epididymal adipose tissue. Data are expressed as mean ± SD. ***P < .0001 versus HFD group. Color images are available online.

The VCO combined with HFD increased the adipocyte area in the retroperitoneal (Fig. 4A, C; *P* < .0001) and epididymal (Fig. 4B, D; *P* < .0001) tissues when compared with the HFD without VCO. These findings indicate that VCO supplementation induced a significant level of adipocyte hypertrophy in retroperitoneal and epididymal adipose tissue of HFD-fed rats.

### Gene expression of adipokines and inflammatory markers in the adipose tissue

**FIG. 5.**
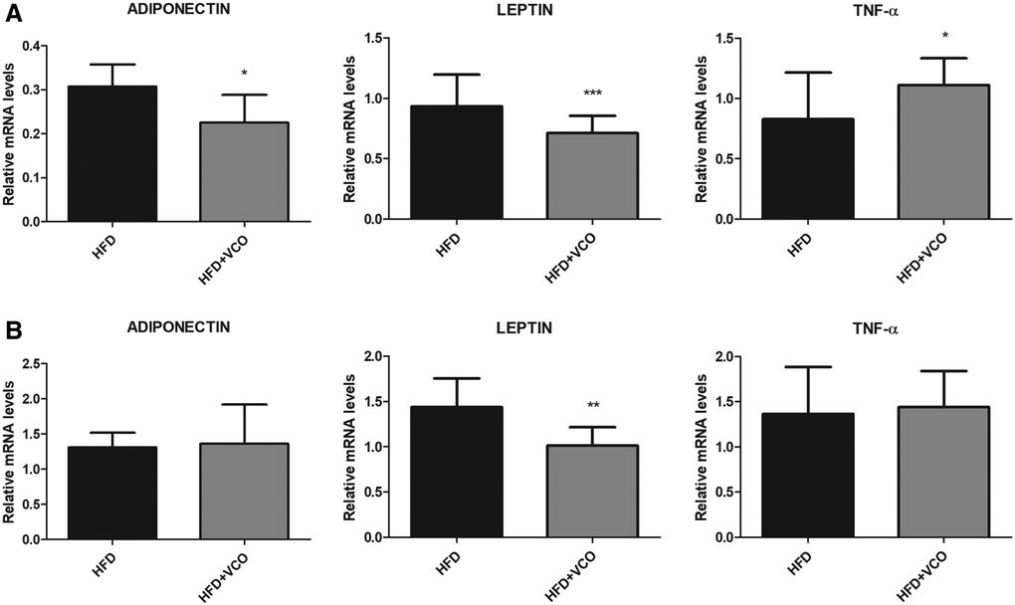
Analysis of Adiponectin, Leptin, and TNF-*α* mRNA expression in adipose tissues of rats fed with HFD or HFD+VCO supplementation. The expression levels of mRNA were analyzed by qRT-PCR in the different experimental groups. In **(A)** retroperitoneal adipose tissue; in **(B)** epididymal adipose tissue. The data were normalized against GAPDH and YWHAZ transcript levels. Data are expressed as mean ± SD. *P < .05, **P < .01, ***P < .0001 versus HFD group. mRNA, messenger RNA; qRT-PCR, quantitative real-time polymerase chain reaction; TNF-*α*, tumor necrosis factor alpha.

Adiponectin gene expression was significantly decreased in retroperitoneal adipose tissue in HFD+VCO group (Fig. 5A; *P* < .05) compared with HFD group. Moreover, leptin gene expression was reduced in retroperitoneal (Fig. 5A; *P* < .0001) and epididymal adipose tissue in HFD+VCO group (Fig. 5B; *P* < .01) compared with HFD group. Tumor necrosis factor alpha (TNF-*α*) gene expression was significantly increased in retroperitoneal adipose tissue in HFD+VCO group (Fig. 5A; *P* < .05) compared with HFD.

## Discussion

In the present study, we investigate the effects of VCO associated with HFD on biochemical, inflammatory, and oxidative stress parameters using the rat as a model. In addition to important metabolic alterations, our findings provide evidence that the HFD+VCO also induced (1) fat deposition in the liver; (2) lipid peroxidation; and (3) adipocyte hypertrophy, which were associated with adipose inflammatory gene expression.

In contrast to that which has been promoted by the media, the use of coconut oil did not induce weight loss. Food intake and weight loss have not been consistently described when VCO is supplemented with different diets and different animal models.^2,31–33^ In this study, VCO supplementation was not associated with higher satiety, since dietary intake was higher in the HFD+VCO group. Furthermore, the animals in the HFD+VCO group also showed an increase in weight gain. Consumption of a diet with a high amount of fat can alter the patterns of food consumption, leading to an increase in palatability-induced intake.^34^ In addition, fat intake has a weak satiety-signaling property.^35^ Considering that both diet and VCO are rich in SFA, we believe that the increase in dietary intake was due to palatability and, consequently, led to an increase in weight gain.

We observed that HFD+VCO decreased glucose levels. This effect may be related to improved insulin sensitivity and glucose tolerance, probably due to the high content of MCFA in the coconut oil.^36,37^ In addition, increasing availability of fatty acids and triglycerides through diet and VCO may have altered the use of glucose in mitochondrial oxidative metabolism.^38^ Metabolic changes related to glucose metabolism and insulin resistance are closely associated with increased dietary fat intake.^39–41^

In the present study, the supplementation of VCO associated with HFD was not able to improve the serum lipid profile and increased LDL-cholesterol level. Inexplicably, total cholesterol level was reduced, whereas LDL-cholesterol level was increased when VCO was present in HFD. One explanation may be that changes in lipid biochemistry, intramolecular disorder, inflammatory, and oxidative stress may have altered the LDL metabolism through disruption of (1) enzymes that modulate the intracellular synthesis of cholesterol; (2) enzyme transcriptions that regulate LDL within the cell; (3) LDL receptor synthesis; (4) LDL degradation; or (5) LDL feedback mechanism. Independently of the mechanism, this finds support that dietary fats and oils can influence the metabolism of lipids and increase the chance of CVD.^42^ Studies have suggested that incorporation of coconut oil into a diet improves serum lipid profile^18,33,43,44^; however, findings concerning this effect are still contradictory. Coconut oil is also associated with increased levels of cholesterol,^45,46^ LDL cholesterol,^11^ and the chance of CVD due to its high SFA concentration.^31,47^

Our results demonstrated that rats fed with HFD and supplemented with VCO have high levels of plasma lipid peroxidation. In addition, as a compensatory response to oxidative damage caused by chronic exposure to HFD and VCO, the animals presented an increase in the activity of antioxidant enzymes SOD, CAT, and GPx. Long-term consumption of HFD causes damage at the cellular and molecular levels and triggers an oxidative stress.^38,48^ Lipids are essential for biological membrane components and represent primary targets for reactive oxygen species (ROS) attack. The increase of ROS and high levels of LDL cholesterol exposes this lipoprotein to oxidation, resulting in the peroxidation of the lipid constituents of the lipoprotein, leading to foam cell formation and favoring vascular adhesion and atherogenesis.^49^

The histological findings of the present study showed that the VCO combined with HFD did not increase the aortic IMT. The measurement of the aortic IMT is a predictor for detecting the changes of vascular structure that may be an indicator of subclinical atherosclerosis and subsequent CVD risk factor.^50,51^ VCO is rich in SFA and a higher intake of this fat is related to the risk of CVD due to the increase of plasma LDL cholesterol levels.^52^ However, there is currently no clear evidence regarding the effects of VCO on lipids and blood lipoproteins; therefore, coconut oil consumption is not indicated for the reduction of cardiovascular risk.^53^

In our study, HFD+VCO induced liver damage, as evidenced by increased activity of aminotransferases, increased hepatic tissue weight, and higher hepatic cholesterol and triglycerides content. Furthermore, the VCO supplementation increased hepatic lipid accumulation in HFD-fed rats, since there was a greater accumulation of lipids droplets, as revealed by the histopathological analysis. HFD can lead to an excess of circulating fatty acids, which promotes ectopic deposition in nonfatty tissues, increasing lipid deposits in the liver.^54^ Previous studies have shown the association between SFA consumption and hepatic steatosis.^55^

Histological analysis of adipose tissue revealed that VCO combined with HFD induced adipocyte hypertrophy in retroperitoneal and epididymal adipose tissue. Adipocyte size is a determinant factor for the secretion of various inflammatory adipokines, which are links between adipocyte hypertrophy and inflammatory response in adipose tissue.^56–58^ Excess of adiposity results in the dysregulation of adipose tissue-derived secretory factors, which may contribute to the development of various metabolic disorders.^56,58^ In this context, we investigate whether adipocyte hypertrophy induced by VCO supplementation combined with HFD would be associated with adipose inflammation. The gene expression analysis showed that HFD plus VCO caused a significant increase in gene expression of TNF-*α* in retroperitoneal adipose tissue. A high SFA intake has been associated with proinflammatory response and gene regulation involved in inflammatory pathways, such as TNF-*α*, in adipose tissue.^59,60^ In contrast, we observed a reduction in the amount of adiponectin messenger RNA (mRNA) expression, with increasing adipocyte size when VCO was combined with HFD. These results evidence that VCO when associated with HFD induces low-grade inflammation in adipose tissue, altering cytokines production, increasing proinflammatory and reducing anti-inflammatory markers.

In our study, reduction in adiponectin gene expression was accompanied by increase in TNF-*α* gene expression in retroperitoneal tissue of HFD+VCO group. Obesity is associated with a decrease of adiponectin gene expression in adipose tissue and this association can to be linked to the increase of inflammatory status of adipose tissue.^61^ In addition, TNF-*α* is known to reduce adiponectin gene expression.^62^ In addition, since our experimental model was not with obesity or metabolic syndrome, we cannot necessarily expect that the adipocyte is expressing less adiponectin concomitantly with increase leptin. In this sense, we can infer that even in the face of the inflammatory process, the adipocyte was not regulating leptin expression as expected.

Obesity is defined as a condition of excessive fat accumulation in adipose tissue.^63^ The expansion of adipose tissue by hyperplasia and/or hypertrophy produces a high concentration of adipokines, which leads to a chronic inflammatory state in the organism, and interact with a range of process in different organs.^57,64^ Furthermore, obesity-induced inflammation is frequently associated with increased oxidative stress.^65,66^ Hepatic lipid accumulation is a common metabolic complication associated with obesity and is caused by imbalanced lipogenesis and lipid oxidation in the liver. New evidence indicates that the metabolic communication between the liver and adipose tissue can play an important role in causing hepatic fat deposition in the obesity-related disorders.^57^

In summary, VCO associated with HFD leads to increase in diet intake, LDL cholesterol levels, adipose inflammatory gene expression, AST and ALT levels, weight gain, hepatic fat accumulation, lipid peroxidation, and adipocyte hypertrophy. Therefore, recommendations regarding VCO consumption should be made with caution, especially when combined with HFD, considering the risks associated with chronic exposure to SFA.
